# Getting ready for DNA duplication

**DOI:** 10.7554/eLife.51291

**Published:** 2019-09-27

**Authors:** Nina Y Yao, Michael E O'Donnell

**Affiliations:** 1 Laboratory of DNA ReplicationThe Rockefeller UniversityNew YorkUnited States; 2 The Howard Hughes Medical Institute and the Laboratory of DNA ReplicationThe Rockefeller UniversityNew YorkUnited States

**Keywords:** DNA replication, phase separation, intrinsically disordered, ORC, Cdc6, Cdt1, *D. melanogaster*

## Abstract

The discovery of a biomolecular condensate involved in DNA replication has wide-ranging implications.

**Related research article** Parker MW, Bell M, Mir M, Kao JA, Darzacq X, Botchan MR, Berger JM. 2019. A new class of disordered elements controls DNA replication through initiator self-assembly. *eLife* **8**:e48562. doi: 10.7554/eLife.48562

Research into biomolecular condensates – self-contained regions within cells where specific reactions take place – is taking cell biology by storm. Biomolecular condensates do not have membranes, but they are able to keep the proteins and/or nucleic acids involved in a particular reaction separate from the rest of the cell. Biomolecular condensates do not have membranes, but they are able to keep the molecules involved in a particular reaction (usually proteins, but sometimes also nucleic acids) separate from the rest of the cell (Boeynaems et al., 2018; Ditlev et al., 2018). They can be found in both the nucleus and the cytoplasm, and are involved in reactions ranging from signal transduction to RNA metabolism. The nucleolus, a structure in the nucleus where ribosomes are synthesized, is an example of a biomolecular condensate (Ahmad et al., 2009).

The physics behind the formation of biomolecular condensates is not fully understood but it often involves at least one protein with an intrinsically disordered region that does not fold into a specific 3D structure, and a process called phase separation (which, as its name suggests, involves regions with different physical properties separating from each other). The molecules required for the formation of condensates are called scaffolding factors, while the molecules encapsulated inside are known as clients. A given condensate typically contains only those clients involved in the relevant reaction.

Now, in eLife, Michael Botchan (UC Berkeley), James Berger (Johns Hopkins) and colleagues – including Matthew Parker as first author – report on the discovery of a biomolecular condensate required for the initiation of DNA replication in the fruit fly *Drosophila melanogaster* (Parker et al., 2019). Three proteins – ORC, Cdc6 and Cdt1 – form the scaffold along with DNA, while a hexamer ring protein called Mcm2-7 is the client. During the G1 phase of the cell cycle, the scaffold proteins load two Mcm2-7 hexamers onto the DNA to form the pre-replicative complex, which marks the spot where DNA replication will begin (Figure 1A–C; Bell and Labib, 2016; Bleichert et al., 2017). Later, in the S phase of the cell cycle, other proteins assemble onto the complex, converting the Mcm2-7 hexamers into helicases that unwind the DNA for replication (Figure 1D).

**Figure 1.**
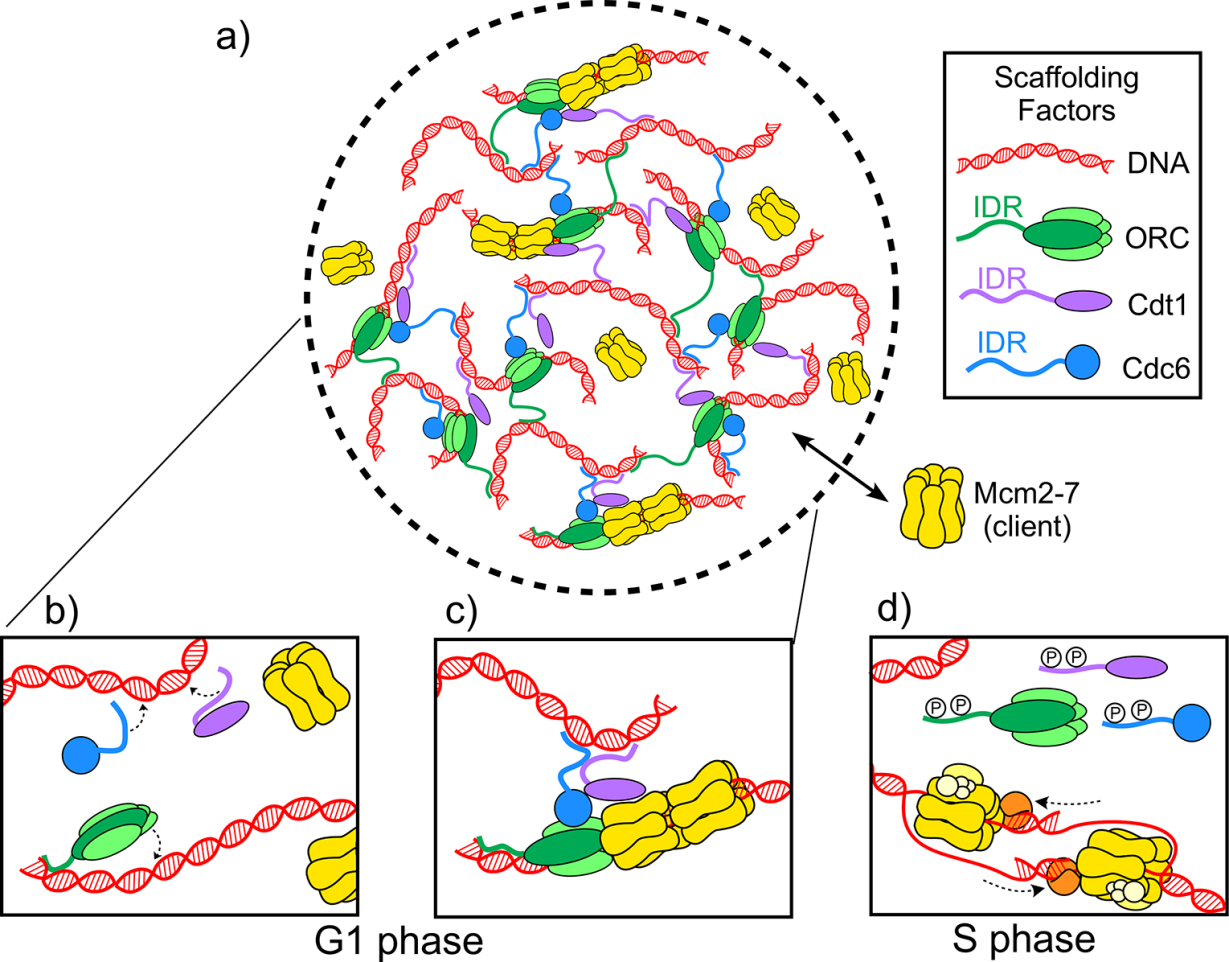
The molecules involved in the initiator biomolecular condensate during the G1 and S phases of the cell cycle. (**A**) Parker et al. studied how three proteins – ORC (green), Cdt1 (purple) and Cdc6 (blue) – work together to help two Mcm2-7 hexamers (yellow) form a pre-replicative complex with DNA (red). (**B**) During the G1 phase of the cell cycle intrinsically disordered regions (IDR) in the origin of replication complex (ORC), Cdc6 and Cdt1 bind a DNA molecule to form a biomolecular condensate. (**C**) The biomolecular condensate forms a scaffold that the Mcm2-7 hexamer can enter, and the pre-replicative complex is formed. (**D**) During the S phase of the cell cycle, the IDRs of the scaffold proteins are phosphorylated (represented by the letter P), so they can no longer form condensates. Mcm2-7 hexamers loaded on the DNA can then interact with other proteins (orange and yellow) to form helicases that unwind the DNA during replication.

When Parker et al. mixed *Drosophila* Cdt1 with DNA, they obtained a turbid solution that formed oil-like droplets under the microscope, a hallmark of biomolecular condensates. The same effect was observed when they mixed *Drosophila* ORC and Cdc6 with DNA. Additionally, Parker et al. found that they could disrupt the formation of these condensates by deleting the intrinsically disordered regions of any of the three proteins involved. On the other hand, Mcm2-7 failed to form a condensate when mixed with DNA. Instead, it acted as a client that could enter the ‘initiator biomolecular condensate’ formed by the scaffold proteins, and once inside, be loaded onto the DNA. Parker et al. demonstrated the selectivity of this condensate by showing that it denied entry to a protein that can form other biomolecular condensates but is not involved in initiating DNA replication.

The initiator biomolecular condensate is required for DNA replication to start, an important step in the cell cycle. Parker et al. found that Cdc6, Cdt1 and ORC could all be phosphorylated on their intrinsically disordered regions by cell cycle kinases, which stopped the formation of the condensate. This interaction between cell cycle kinases and the scaffold proteins links the formation of the pre-replicative complex to the cell cycle.

But why would the cell form a biomolecular condensate for the pre-replicative complex reaction at all? Parker et al. note that the budding yeast, a cell with a relatively small genome, does not form an initiator condensate. Hence, the selection pressure to evolve this particular condensate might lie in the genomic complexity of organisms.

When animal cells divide, DNA duplication starts at specific sites in the genome called origins of replication, where the pre-replicative complexes are assembled. There are multiple origins of replication throughout the genome, and the start of DNA duplication at each one is referred to as ‘firing’. Origins of replication appear to fire stochastically, with some firing early during the S phase of the cell cycle and others later on (Kaykov and Nurse, 2015). But if one takes an eagle’s view, there are indications that the process may happen in a particular order in time and space. For example, origins that fire early sometimes appear in clusters, and certain origins prevent others from firing, suggesting that the origins can communicate with each other (Cayrou et al., 2011; Duriez et al., 2019). Thus, initiator biomolecular condensates might gather distant regions of DNA to form pre-replicative complexes in a cluster, facilitating temporal and spatial organization of replication start sites. The discovery of these condensates therefore represents an important step toward a more complete understanding of DNA replication in complex genomes.
